# Changes in the process of alternative RNA splicing results in soluble B and T lymphocyte attenuator with biological and clinical implications in critical illness

**DOI:** 10.1186/s10020-018-0036-3

**Published:** 2018-06-18

**Authors:** Sean F. Monaghan, Debasree Banerjee, Chun-Shiang Chung, Joanne Lomas-Neira, Kamil J. Cygan, Christy L. Rhine, William G. Fairbrother, Daithi S. Heffernan, Mitchell M. Levy, William G. Cioffi, Alfred Ayala

**Affiliations:** 1 Division of Surgical Research, Department of Surgery, Alpert School of Medicine at Brown University and Rhode Island Hospital, 593 Eddy Street, Providence, RI 02903 USA; 2 Division of Pulmonary and Critical Care, Department of Medicine, Alpert School of Medicine at Brown University and Rhode Island Hospital, Providence, RI 02903 USA; 3 MCB Department, Brown University, Providence, RI 02903 USA

**Keywords:** BTLA, ARDS, RNA splicing, Critical illness

## Abstract

**Background:**

Critically ill patients with sepsis and acute respiratory distress syndrome have severely altered physiology and immune system modifications. RNA splicing is a basic molecular mechanism influenced by physiologic alterations. Immune checkpoint inhibitors, such as B and T Lymphocyte Attenuator (BTLA) have previously been shown to influence outcomes in critical illness. We hypothesize altered physiology in critical illness results in alternative RNA splicing of the immune checkpoint protein, BTLA, resulting in a soluble form with biologic and clinical significance.

**Methods:**

Samples were collected from critically ill humans and mice. Levels soluble BTLA (sBTLA) were measured. Ex vivo experiments assessing for cellular proliferation and cytokine production were done using splenocytes from critically ill mice cultured with sBTLA. Deep RNA sequencing was done to look for alternative splicing of BTLA. sBTLA levels were fitted to models to predict sepsis diagnosis.

**Results:**

sBTLA is increased in the blood of critically ill humans and mice and can predict a sepsis diagnosis on hospital day 0 in humans. Alternative RNA splicing results in a premature stop codon that results in the soluble form. sBTLA has a clinically relevant impact as splenocytes from mice with critical illness cultured with soluble BTLA have increased cellular proliferation.

**Conclusion:**

sBTLA is produced as a result of alternative RNA splicing. This isoform of BTLA has biological significance through changes in cellular proliferation and can predict the diagnosis of sepsis.

## Background

Patients with severe critical illness, such as sepsis and acute respiratory distress syndrome, have severely modified physiology resulting in organ dysfunction. The current definition of sepsis utilizes the SOFA score to standardize the organ dysfunction that results (Singer et al., 2016). As there is more focus on organ dysfunction, more effort is needed to understand basic molecular mechanisms that may influence critical illness and the subsequent organ dysfunction.

It is known that physiologic conditions seen in critical illness, such as hypoxia and acidosis, influence the normal process of RNA splicing (Elias & Dias, 2008). Preliminary data also suggests that models of critical illness result in multiple instances of statistically significant changes in the alternative RNA splicing process and/or the nature/levels of gene products transcribed into mRNA/protein (Monaghan et al., 2017). A better understanding of alternative RNA splicing as it pertains to immune modulating proteins is needed as not only are many of these proteins proposed to be central mediators of pathological process that contribute to organ dysfunction, but as these proteins become therapeutic targets in critical illness more needs to be understood about the impact of alternative splicing impact on their pharmacological impact. In this respect RNA splicing is the proposed mechanism for processing of message for the soluble form of the immune cell-surface co-inhibitory receptor, a.k.a., a checkpoint protein, B and T lymphocyte attenuator (sBTLA).

Lack of the gene for BTLA has been shown to improve mortality in animal models of sepsis (Shubin et al., 2012; Cheng et al., 2016). In addition, humans with increased leukocyte cell surface expression of BTLA are more likely to have sepsis, are at an increased risk of subsequent infections, and have longer hospital lengths of stay (Shubin et al., 2013). In addition, the soluble form has recently been shown to be elevated in sepsis and predict mortality and disease severity (Lange et al., 2017) and alternative RNA splicing is the proposed mechanism for the generation of sBTLA (Elias & Dias, 2008; Kasim et al., 2014). Soluble BTLA has also been implicated in enhanced vaccine response to cancer (Han et al., 2009; Han et al., 2014).

However, although previous work has suggested an important role in critical illness for sBTLA, there was no mention of how this isoform is produced or its biologic relevance. In this respect, PD-1 is a similar immune cell-surface co-inhibitory molecule/checkpoint protein inhibitor with similar effects in critical illness (Huang et al., 2009; Monaghan et al., 2012a; Monaghan et al., 2012b). PD-1 also has a soluble form that is increased with a biologic impact and this isoform is due to alternative RNA splicing (Monaghan et al., 2016). Here we propose that sBTLA is produced as a result of alternative RNA splicing and the soluble isoform has both biologic and clinical importance.

## Methods

### Collection of samples from humans

Samples from humans were collected from patients admitted to the medical intensive care unit (MICU) at Rhode Island Hospital. Patients were considered to have sepsis if they had corresponding ICD-10 coding on admission, attending physician documentation of sepsis, septic shock in their problem list with evidence of hypo-perfusion (lactate > 2 mmol/L, systolic blood pressure < 90 mmHg, mean arterial pressure < 65 after 30 cm^3^/kg crystalloid bolus within 3 h of identification), suspected or documented infection with evidence of at least one organ failure as defined by the SOFA score. (Singer et al., 2016) Patients were excluded due to malignancy, trauma within 30 days, pulmonary fibrosis, known recent antibiotic use (within last week of admission), prisoners, pregnancy, age less than 18 years old and immune-compromised state. Patients were enrolled upon arrival to the ICU or within 24 h of developing sepsis in the ICU. Consent was obtained and the IRB approved this study (IRB# 4159–14).

### Collection of samples from mice with critical illness

C57BL/6 male mice (The Jackson Laboratory, Bar Harbor, ME) between 10 and 12 weeks of age were used. Critical illness was induced in the mice by hemorrhage (non-lethal shock) followed by cecal ligation and puncture (CLP) (Monaghan et al., 2012a; Ayala et al., 2002; Lomas-Neira et al., 2006; Perl et al., 2007; Thakkar et al., 2011). The control group was sham hemorrhage followed by sham CLP (Sham-Sham). All experiments were done according to guidelines from the National Institutes of Health (Bethesda, MD) and were approved by the Rhode Island Hospital animal use committee (AWC#: 0206–15).

### Measurement of sBTLA

Blood from mice and humans was centrifuged at 10,000×g at 4°C for 10 min, the serum layer was isolated, and red blood cells were lysed with 1 mL double distilled water with 0.037 g EDTA (Invitrogen, Carlsbad, CA), 8.26 g NH_4_Cl (Sigma, St. Louis, MO), and 1 g KHCO_3_ (Sigma, St. Louis, MO). In humans sBTLA was measured using a multiplex analysis with the Thermo Fischer multiplex kit (Waltham, MA). In mice the soluble level of BTLA was measured in the serum using the BTLA ELISA kit (Cusabio, College Park, MD). A bronchial alveolar lavage (BAL) sample was collected from the mice after euthanasia as previously done (Monaghan et al., 2016). These samples were tested for BTLA using the same kit for the serum (Cusabio, College Park, MD). The detection of soluble BTLA was done in samples that were free of cellular components to minimize the amount of membrane bound BTLA that may be detected in the samples and this is in line with previous work regarding soluble immune modulating proteins (Lange et al., 2017; Monaghan et al., 2016).

### Ex vivo experiments

Splenocytes were harvested from either mice with severe critical illness (hemorrhage/CLP) or sham controls as previously described (Monaghan et al., 2016). In brief, after the mice were euthanized with CO_2_ the spleens were harvested and then crushed between two slides to liberate the cells into 10 mL sterile PBS. Red blood cells were lysed with sodium chloride. Splenocytes were cultured with DMEM (ThermoScientific, Waltham, MA) with 10% fetal calf serum (Atlantic Biologicals, Miami, FL) and 0.1% gentamycin (Sigma, St. Louis, MO).

BTLA fusion protein (R&D Systems, Minneapolis, MN) at 1000 ng/mL or 10,000 ng/mL or control was then used as an additive to the culture of splenocytes (as above) from mice with critical illness compared to mice who underwent sham hemorrhage and sham CLP as previously described (Monaghan et al., 2016). This fusion protein was selected as it mimics the binding of the extracellular portion of BTLA and will be added to culture in conditions similar to the soluble form. All cells were cultured for 72 h. At that point, proliferation was assessed using the CyQuant assay (ThermoScientific, Waltham, MA). Supernatants were collected and analyzed for the production of cytokines using a multiplex analysis with the Mouse Th 1/2/9/17 panel 17 multiplex kit (Thermo Fischer, Waltham, MA) per the manufacturer instructions on a Luminex machine (Austin, TX).

### Isolation of RNA and RNA sequencing

Samples were collected as whole blood from mice with critical illness induced by hemorrhagic shock followed by sepsis via CLP (IACUC approved) as described above. RNA was extracted using the MasterPure Complete DNA/RNA Purification kit (epicenter, Madison WI) followed by the Globin Clear Kit (ThermoScientific, Waltham, MA). RNA was then sent to Gene Wiz for sequencing as 1400 ng RNA in 40uL of fluid.

### Assessment of RNA splicing

The raw RNA sequencing data first underwent quality control with MultiQC (Ewels et al., 2016). Using rMATS, alternative RNA splicing events were identified, in addition to an assessment of gene transcription (Li et al., 2017; Shen et al., 2012; Shen et al., 2014). Significant alternative RNA splicing events in the BTLA gene were then further studied to predict the protein outcome for those events.

### Statistical analysis

Data was analyzed using SigmaPlot 10.0 (Systat Software, San Jose, CA) or code contained within rMATS. Paired t-tests or rank sum analysis were done when two groups are compared. Multiple groups were compared by or two-way ANOVA with Holm-Sidak correction. Receiver operator characteristic curves were fit to use sBTLA level to predict sepsis diagnosis. Graphs are displayed as mean or median with error bars representing the standard deviation. Alpha was set to 0.05.

## Results

**Fig. 1:**
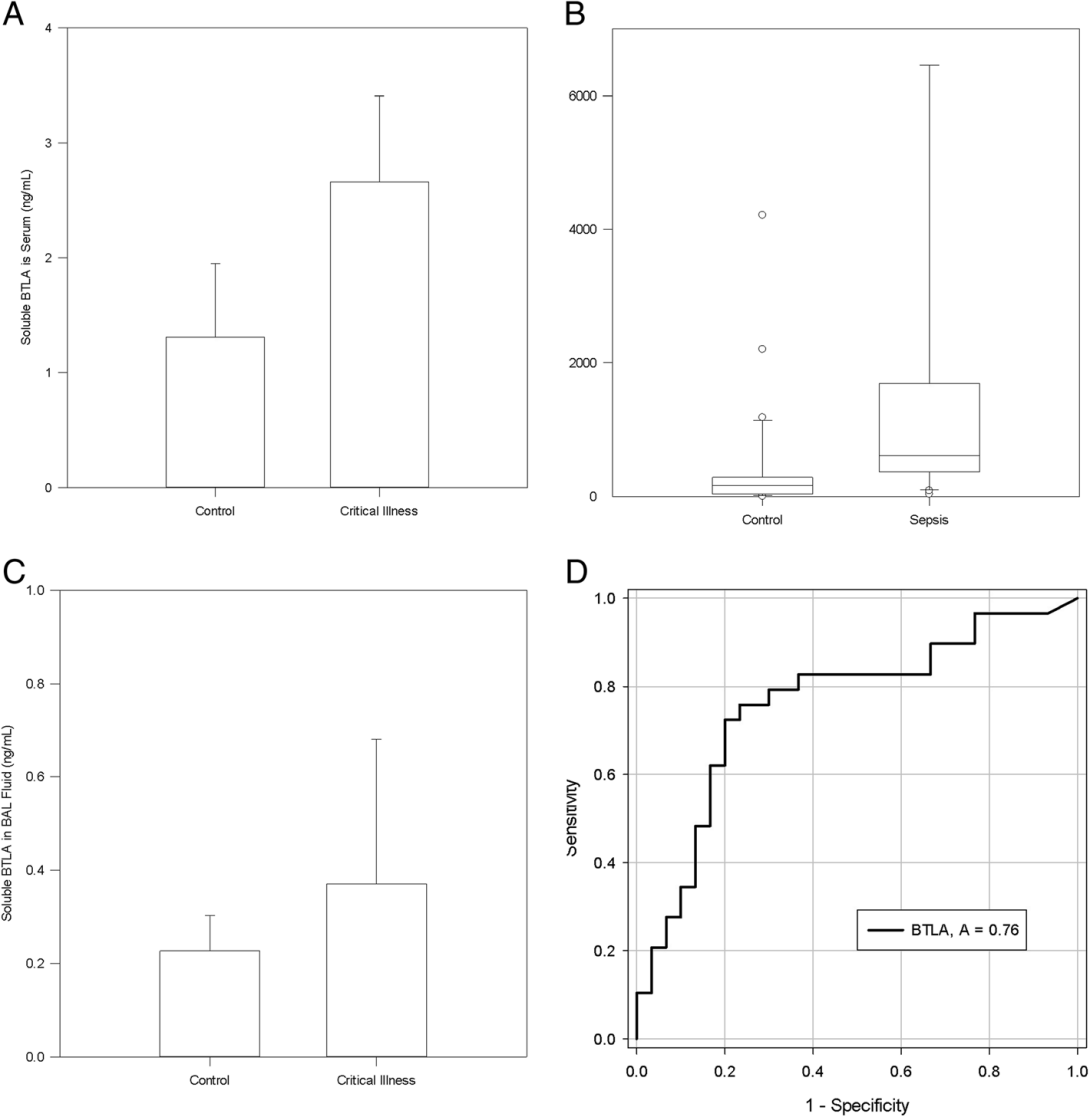
– Soluble BTLA in Critical Illness: SBTLA is increased in the serum of mice (Figure **a**, *p* = 0.0037) and humans (Figure **b**, *p* < 0.001), but no differences were seen in the BAL fluid of mice (*p* = 0.476, Figure **c**), and changes in critically ill patient levels of sBTLA on day 0 of hospital admission predict the diagnosis of sepsis (Figure **d**)

Soluble BTLA levels were assessed in the lungs, BAL fluid and serum of mice with severe critical illness induced by hemorrhagic shock followed by septic shock. Mouse sBTLA increased in the serum in critical illness as compared to controls (2.66 ng/mL vs 1.208 ng/mL, *p* = 0.0037, Fig. 1a), but no differences were seen in the BAL fluid (0.371 ng/mL vs. 0.227, *p* = 0.476, Fig. 1c) or the lung samples (40.077 ng/mL vs. 43.086 ng/mL, *p* = 0.519).

Humans were recruited from a medical intensive care unit after consent was obtained, 30 had a diagnosis of sepsis (Singer et al., 2016) and 30 were non-sepsis ICU controls. 5 patients in the sepsis group and 1 patient in the control group died during the hospital stay despite similar ages between the groups (sepsis 60, control 56). Human sBTLA in the blood increased in sepsis vs ICU controls (557.030 pg/mL (255.370–1485.693) vs 158.350 (33.233–284.831), *p* < 0.001, Fig. 1b). In addition the level of sBTLA can predict sepsis on hospital day 0 (ROC curve area 0.7598, 95% CI 0.6326 To 0.8869, *p* = 0.0006111815643966, Fig. 1d).

**Fig. 2:**
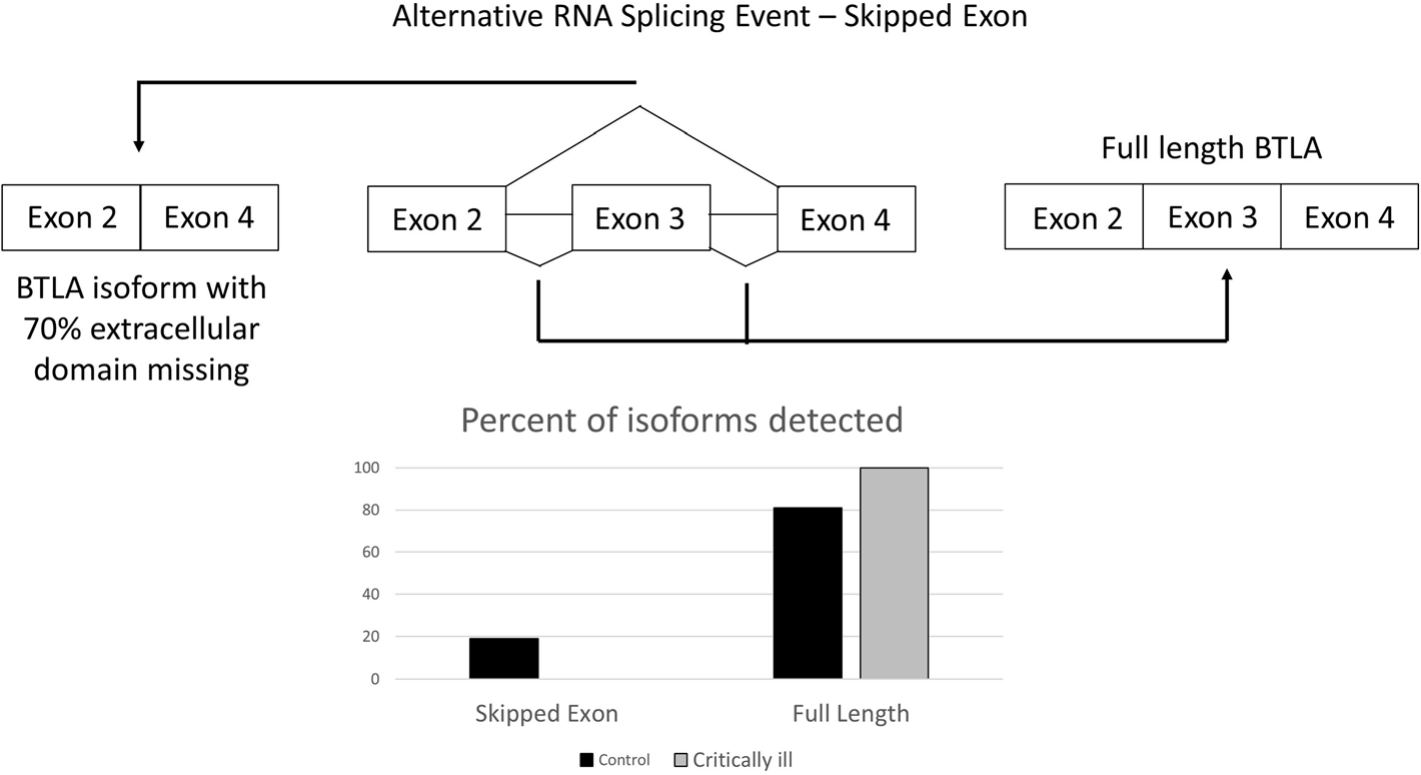
– Frequency of Skipped Exon in BTLA in critically ill mouse model: The skipping of exon 3 is significantly different between critically ill mice (19%, *n* = 3) and healthy sham controls (0%, n = 3) (FDR =0.0000471413836931, bar graph to the left). The full length isoform is seen 81% of the time in the control mice and 100% of the time in the critically ill mice (bar graph to the right)

**Fig. 3:**
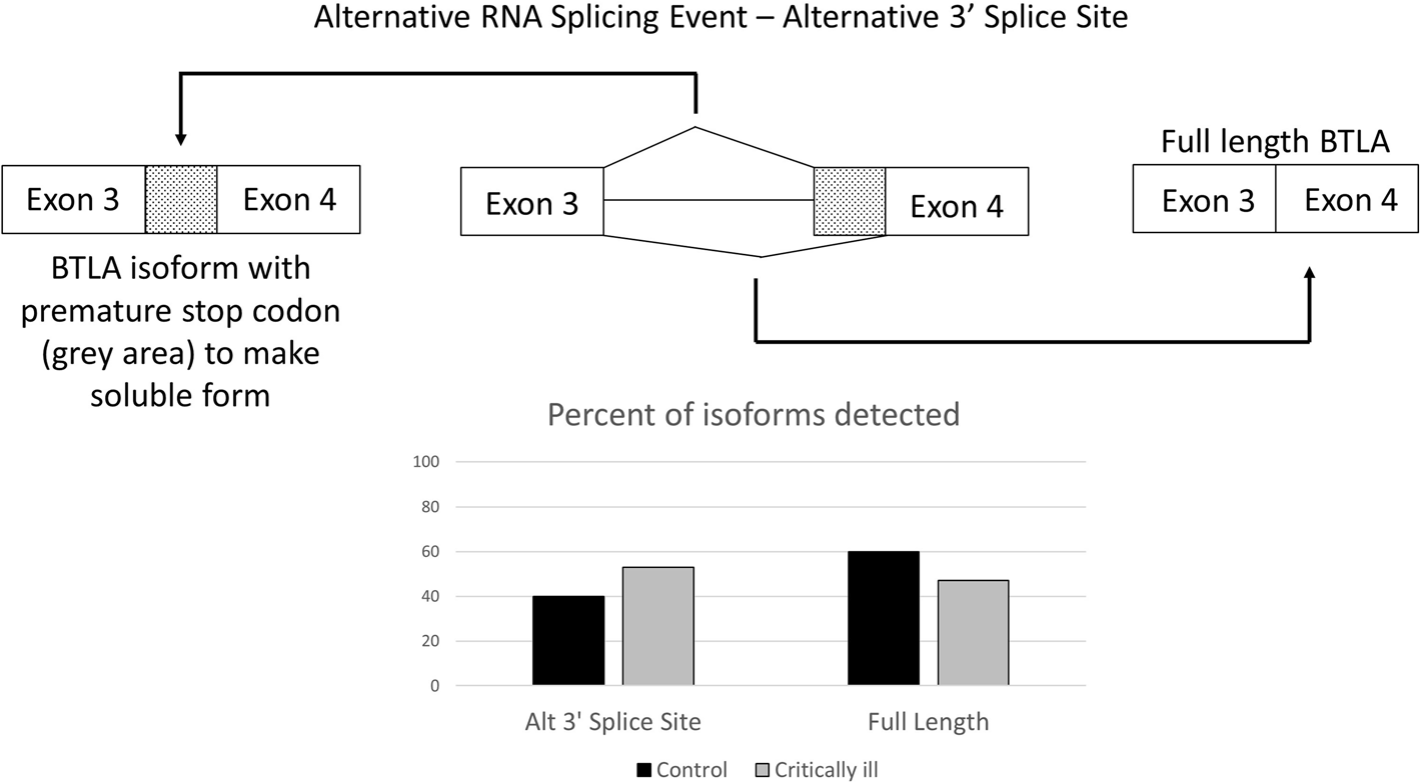
– Alternative 3’ Splice Site of BTLA: The percentage of an alternative 3′ splice site at exon 4 is significantly different between critically ill mice (40%, n = 3) and healthy controls (53%, n = 3) (FDR =0.0000429857901449, bar graph to the left). The full length isoform is seen 60% of the time in the control mice and 47% of the time in the critically ill mice (bar graph to the right)

In order to assess the potential mechanism by which sBTLA is produced, RNA from the blood of mice with critical illness and controls was sent for deep RNA sequencing to look for alternative RNA splicing. From this sequencing it was found that the while the extent of overall gene expression of BTLA was similar between the two groups, there were changes in two significant splicing events noted. A skipped exon event was noticed in which exon 3 is skipped (Fig. 2). Exon three encodes much of the extracellular domain of BTLA, and as a result, this event leads to the loss of 70% of the extracellular domain. In the control mice, the exon is skipped 19% of the time, but in critical illness, the exon is never skipped (FDR =0.0000471413836931). A second significant splicing event predicts the production of sBTLA. In this event there is an alternative 3′ splice site before exon 4 (Fig. 3). This alternative 3′ splice site results in a premature stop codon so only exons 1–3 are translated. This protein with exons 1–3 could be the soluble form as the transmembrane and intracellular portions are not translated with this isoform. In the control mice the alternative 3′ splicing site occurs 40% of the time, but in critically ill mice, this level increases to 53% (FDR =0.0000429857901449).

**Fig. 4:**
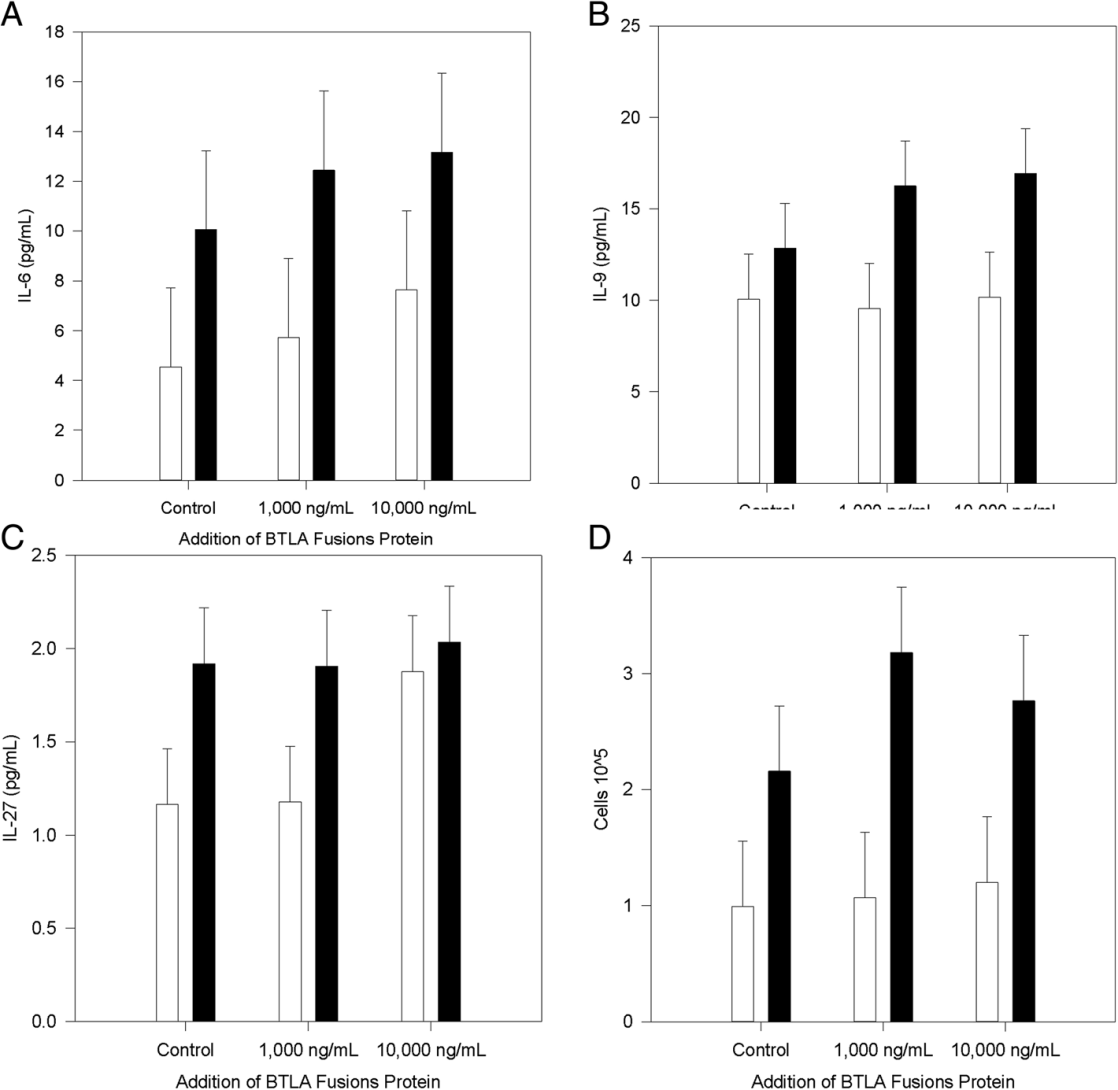
– Ex Vivo Experiments with BTLA Fusion Protein: When cells came from mice with critical illness there were increased levels of IL-6 (*p* = 0.41, Figure **a**), IL-9 (*p* = 0.019, Figure **b**), IL-27 (*p* = 0.044, Figure **c**) and cellular proliferation (*p* = 0.004, Figure **d**). The addition of 1000 ng/mL of BTLA fusion protein also increased cellular proliferation (*p* = 0.021, Figure **d**)

Since sBTLA is increased in sepsis/critical illness and results from the alternative RNA splicing analysis implied that nature of the process had changed, experiments were undertaken to assess a biological significance of sBTLA. Splenocytes from mice with critical illness or controls were cultured with a BTLA fusion protein and cytokines and cellular proliferation were assessed. In the supernatant of the cells there was no significant difference in the levels of granulocyte-monocyte colony stimulating factor (GM-CSF), interferon (IFN)-gamma, interleukin (IL)-1beta, IL-2, IL-4, IL-5, IL-12p70, IL-13, IL-18, IL-10, IL-17a, IL-22, IL-23, or tumor necrosis factor (TNF)-alpha based upon the source of the cells (critically ill mice or control) or level of BTLA (1000 ng/mL vs 10,000 ng/mL). Cells from mice with critical illness produced significantly more IL-6 (Diff of means 5.919, *p* = 0.41, Fig. 4a), IL-9 (Diff of means 5.410, *p* = 0.019, Fig. 4b) and IL-27 (Diff of means 0.549, *p* = 0.044, Fig. 4c), but there was no influence on the sBTLA fusion protein. Cellular proliferation was increased in cells from mice with critical illness (Diff of Means 1.615, *p* = 0.004, Fig. 4d). Although there were no changes in cytokine production during the co-culture with the BTLA fusion protein across the two cell groups (critically ill and controls), the 1000 ng sBTLA/mL resulted in increased proliferation when comparing cells from mice with critical illness (Diff of means 2.112, *p* = 0.021) to controls.

## Discussion

In this study we have again found that sBTLA levels are increased in humans with sepsis, but also in a murine model of critical illness (Fig. 1a and b) and this is inline with previous clinical studies of sBTLA (Lange et al., 2017). Further, we show that the rise sBTLA, on hospital day 0, was able to predict the diagnosis of sepsis (Fig. 1d) with prognostic capacity close to SOFA and other soluble co-inhibitory receptors/checkpoint proteins recently reported (Singer et al., 2016; Banerjee et al., 2018). Previous work has focused on the influence of membrane bound BTLA relative to outcomes in the murine model and ICU patients (Shubin et al., 2012; Shubin et al., 2013; Shubin et al., 2011) as assessed by flow cytometry. Assessing sBTLA is easier as it can be accomplished via ELISA of the serum and as such could be a novel biomarker for sepsis with a quicker throughput compared to flow cytometry or bacterial cultures (Shao et al., 2015; Sherwood & Hotchkiss, 2013).

However, for a truly impactful, beyond its potential merits as a biomarker, an understanding of the mechanism by which it is not only produced, but its ultimate function is essential in order to gain insight into pathological potential as well as into clinical/pharmacological scenarios where the levels are not in line with expectations or if BTLA is to be a therapeutic target (Patil et al., 2017). This study has showed that sBTLA is likely the result of changes in the nature but not the extent of the alternative RNA splicing process in response the combined insults of shock and sepsis in mice (Figs. 2 and 3). Importantly, alternative RNA splicing can also explain why previous work has shown an increase in membrane bound levels (Shubin et al., 2012; Shubin et al., 2013; Shubin et al., 2011), but we detected no difference in level of gene expression in our samples. Fig. 2 demonstrates how skipping an exon in the control animal’s results in a relative decrease in the membrane bound BTLA identified because 70% of the extracellular portion is removed, and therefore this protein may not be seen by antibodies directed to the extracellular portion. In addition, alternative splicing has been shown to result in lack of translation to a protein without the creation of a new premature stop codon and this isoform may be degraded (Martinez-Nunez & Sanford, 2016).

Again, alternative RNA splicing can explain how more sBTLA is observed despite similar levels of gene expression. Alternative RNA splicing has been suggested as a mechanism for the production of other membrane bound immune checkpoint proteins such as PD-1 and CTLA-4 (Monaghan et al., 2016; Rossille et al., 2014; Ward et al., 2014). In the current study, the soluble form is created as a result of a premature stop codon from an alternative 3′ splice site (Fig. 3). Although previously stated mechanisms of degradation are possible (Martinez-Nunez & Sanford, 2016), the protein levels seen in fig. 1a and b support the production of the soluble form. However, in order to truly know if an alternatively spliced isoform is translated to a gene advanced techniques utilizing mass spectroscopy (Ivanov et al., 2018) would be needed since the antibodies to proteins may not detect the novel isoform.

The creation of sBTLA via alternative splicing would be novel; however, sBTLA also has some biological function. Despite the testing for numerous cytokines in the supernatant of splenocytes from critically ill mice or controls, few were influenced by critical illness (Fig. 4a, b, c) and none were influenced by the presence of the BTLA fusion protein in the media. Despite no changes in cytokine production from the BTLA fusion protein, it did cause an increase in cellular proliferation at the 1000 ng/mL level; this is the level most in line with the in vivo level detected here. Increase in proliferation shows a biological significance of this protein, but its mechanism is not completely clear. However, sBTLA may explain some differences seen in experiments conducted previously. Lack of BTLA gene expression has reported to reduce septic mortality in murine models (Shubin et al., 2012; Shubin et al., 2013), however, addition of BTLA antibody did not improve septic mouse survival (Cheng et al., 2016). The lack of survival benefit from antibody addition may be due to interference of sBTLA, which we have shown to be increased in this model, particularly if the antibody is directed against the extra-cellular portion. In addition a thorough understanding of the soluble/membrane bound interaction and target of potential antibodies is critical as these checkpoint inhibitors are therapeutic targets (Patil et al., 2017). Another potential reason for limited results as it pertains to cytokine production is the heterogeneous nature of splenocytes. BTLA and its ligand HVEM are located on many cells and results could be minimized due to this heterogeneous cell populations.

## Conclusions

sBTLA is increased in human and murine models of critical illness, is produced as a result of a change in the nature but not the extent of alternative RNA splicing, has biological significance in altering cellular proliferation, and can predict the development of sepsis in in critical ill patients.

## Abbreviations

BAL: bronchial alveolar lavage
BTLA: B and T Lymphocyte Attenuator
CLP: cecal ligation and puncture
Interferon: IFN
Interleukin: IL
MICU: medical intensive care unit
